# Iodoform in Diabetes

**Published:** 1882-09

**Authors:** 


### Iodoform in Diabetes.

The last drug introduced into the treatment of diabetes mellitus is iodoform, which Prof. Moleschott, in a recent communication to the Academy of Medicine at Rome, states he has found to be very beneficial in five cases of that disease. The quantity of sugar excreted rapidly diminished in all cases so treated.

Small doses are sometimes productive of good results, but as much as forty and fifty centigrammes may be administered daily with impunity. The Professor employs cumarin—the odoriferous principle of the Tonquin bean—to overcome the unpleasant smell of iodoform. He prescribes: iodoform, 1.0, extract of lettuce, 1.0, cumarin, 0.1; to be made into twenty pills with powdered gum arabic, and to proceed from one pill twice to two pills four times in the twenty-four hours.—*Lancet.*

